# Disease burden associated with influenza activity at the population level

**DOI:** 10.1017/S0950268826101320

**Published:** 2026-04-06

**Authors:** Aaron C. Miller, Daniel Erik Boonstra, Joseph E. Cavanaugh, Constantina Boikos, Tianyan Hu, John McLaughlin, Timothy Weimken, Verna Welch, Philip M. Polgreen

**Affiliations:** 1 Internal Medicine, The University of Iowa Roy J and Lucille A Carver College of Medicine, USA; 2 Biostatistics, The University of Iowa College of Public Health, USA; 3 Pfizer Canada ULC, Canada; 4 Pfizer Inc, USA

**Keywords:** acute bronchitis, cost, influenza, otitis media, sinusitis

## Abstract

Influenza increases the risk of secondary diseases, but other than pneumonia, many of these diseases (e.g., sinusitis, otitis media, acute myocardial infarctions) are not consistently considered in estimates of influenza burden. We used the Merative Marketscan database (2001–2019) and time-series methods to identify age-specific categories of diseases that were temporally associated with patterns of influenza activity. Next, we estimated hypothetical reductions in the incidence and costs of these diseases if influenza incidence were reduced. Of 282 different disease categories evaluated, 23 (8.2%) were strongly associated with influenza (e.g., acute bronchitis, otitis media, myocardial infarctions, sinusitis, COPD) in at least one age group. For example, we estimated a 20% decrease in peak influenza incidence could decrease acute bronchitis cases by 6.5% and pneumonia cases by 5.3%, corresponding to a $1.6 billion reduction in healthcare costs. Excluding secondary diseases associated with influenza may lead to substantial underestimates of influenza’s burden and costs.

## Background

Influenza is a major cause of morbidity and mortality worldwide [1]. In the U.S., an estimated 120000–710000 hospitalizations and 6300–52000 deaths are attributable to influenza each year [2]. Common symptoms of influenza include fever, cough, headache, sore throat, fatigue, myalgias, and rhinorrhoea [3, 4]. However, severe cases of influenza can result in respiratory failure and death [5]. While infection severity varies across seasons and circulating influenza strains, certain populations (e.g., older adults), and people with comorbidities (e.g., diabetes, chronic pulmonary diseases) may experience more severe disease [4, 6].

Beyond the classic influenza-related symptoms, influenza also increases the risk of secondary diseases. While some estimates of influenza burden include secondary cases of pneumonia [7], influenza also increases the risk for other bacterial infections such as otitis media and sinus infections [8, 9]. Furthermore, some non-infectious diseases may also be associated with influenza (e.g., myocardial infarctions, COPD exacerbations) [10, 11]. These non-infectious diseases follow a seasonal pattern similar to influenza, making it difficult to assert causal relationships. However, the 2009 influenza pandemic provided a natural experiment in that the pandemic peaked months prior to the typical winter peak of influenza. This out-of-season peak was associated with increased hospital admissions for acute myocardial infarctions [12], COPD [13], and asthma [14], highlighting the potential for influenza to elevate the risk of secondary diseases.

If influenza is associated with diseases beyond those typically considered, the overall burden of disease attributable to influenza, as well as the benefits of preventive measures, will be underestimated. Accordingly, there is a critical need to develop more comprehensive disease-burden estimates for influenza to understand and communicate the benefits of preventive interventions for influenza. The primary objective of this research is to identify diseases that may be attributable to influenza without starting with a pre-specified list of potential diseases. Our secondary objective was to estimate the degree to which healthcare utilization and costs may be reduced if influenza rates could be decreased.

## Methods

### Design, setting, and population

We conducted an ecological study of the association between monthly influenza incidence and other related conditions across time, using the Merative MarketScan Research Databases, a large observational database of healthcare claims. Specifically, we used the Commercial Claims and Encounters and Medicare databases, which contain healthcare claims for inpatient, outpatient, and emergency department visits along with outpatient prescription medications (2001–2019).

### Influenza and secondary disease outcome measures

We built time series of the monthly incidence of influenza and secondary diseases for the following age groups: < 2, 2–4, 5–11, 12–17, 18–64, and ≥65 years. To construct the influenza series, we used the following influenza-specific diagnosis codes: ICD-9-CM codes 487.X, 488.X and ICD-10-CM codes J09.X, J10.X, and J11.X. We identified visits in the outpatient or inpatient setting, regardless of the diagnosis order (e.g., primary or secondary). These data were then aggregated at a monthly level to compute the number of enrolees with an influenza diagnosis each month. This temporal scale works well with our proposed computational method [12, 13, 15].

To identify the secondary disease incidence temporally associated with influenza, we considered all diagnosis codes recorded in inpatient and outpatient settings, rather than starting with a pre-specified list of diseases known to be associated with influenza. By reviewing all possible diagnoses related to influenza, we aimed to uncover new links that could guide future research. Specifically, we grouped diagnosis codes into 282 meaningful disease categories using the Agency for Healthcare Research and Quality’s Clinical Classification Software (CCS) groupings [16]. We then computed the monthly incidence of each CCS category by age group.

For each age stratum, we computed the number of enrolees each month with the corresponding diagnoses. To account for enrolment changes in the composition of the MarketScan cohort across time (e.g., changes in the distribution of age groups), we then divided this value by the average number of enrolees in the corresponding month.

### Statistical analysis

To model each age-stratified incidence series, we used a structural (state-space) time-series model [17, 18] that allowed us to decompose each series into three meaningful additive components: a long-term trend component, a recurring seasonal component, and a ‘local component’. The long-term trend characterizes the linear and non-linear incidence rate over time and describes the general trajectory of the series. The seasonal component estimates the seasonal cycles, defined as regular fluctuations that recur each year. The local component represents residual error in addition to an anomaly process that characterizes local temporal correlation and captures deviations from the baseline incidence level that arise from atypically high or low incidence.

### Identifying healthcare utilization associated with influenza activity and secondary diseases

To determine which diseases might occur secondary to influenza for each age group, we fit a structural time-series model to each incidence series. We then identified series that were correlated with influenza at both the global and local levels. Specifically, we sought series that were seasonal, peaking in the winter, and had a similar local component. To do so, we evaluated different correlation thresholds as potential cut-offs for which disease series to include. We selected a cut-off that captured disease series that were clinically plausible, without selecting conditions that could not be plausibly linked to influenza. We deemed a global correlation of 0.6 and a local correlation of 0.2 to be ‘clinically meaningful’ for inclusion. While these correlation coefficients were subjectively determined, they were selected with the goal of identifying secondary diseases based on prior reports of diseases associated with influenza [6, 12–14].

We then used a time-series regression model to estimate the hypothetical reduction in the incidence of the secondary diseases if influenza decreased. Specifically, we explored a range of potential impacts by considering a reduction in the maximum mean monthly incidence (i.e., peak month) of influenza by 20% and 60% for each age group. This range was meant to explore a range of potential reductions that could be achieved through improvements in vaccine effectiveness, increased uptake of influenza vaccines and treatments, or non-pharmaceutical interventions [19]. We evaluated the decrease in healthcare utilization in terms of both a reduction in influenza-specific visits as well and a commensurate reduction in the secondary conditions identified. Supplementary Appendix Methods 1 provides additional details about our structural time-series models, clustering approach, and the healthcare utilization associated with influenza activity.

### Estimating attributable healthcare visit costs

We evaluated the direct medical costs that would be associated with a decrease in influenza incidence (and corresponding secondary disease burden identified via the previous step). We modelled a reduction in influenza incidence during the peak month using the same targeted reduction rates of 20% and 60% for each age group. Because of the overlap between secondary diseases that may co-occur in the same healthcare encounter (e.g., a visit where both influenza and pneumonia were diagnosed) and because a specific condition, such as pneumonia, may involve multiple healthcare encounters, it is not reasonable to additively combine independent estimates of healthcare costs for each condition. To account for this type of multiplicity, where a patient may have multiple secondary conditions attributable to a single influenza event, we used a simulation model to bootstrap these attributable costs. Specifically, the model (1) aggregated patient visits at a monthly level, (2) independently drew visits for each condition based on estimated reduction rates (described above) and reduced to the set of distinctly drawn patient visits (i.e., removing duplicate visits drawn for multiple conditions), and (3) summarized healthcare costs for selected visits. Supplementary Appendix Methods 2 provides additional details about this procedure. This study was deemed non-human-subjects research by the University of Iowa’s Institutional Review Board (HawkIRB). The data that support the findings of this study are available from Merative. Restrictions apply to the availability of these data, which were used under license for this study.

## Results

**Figure 1.**
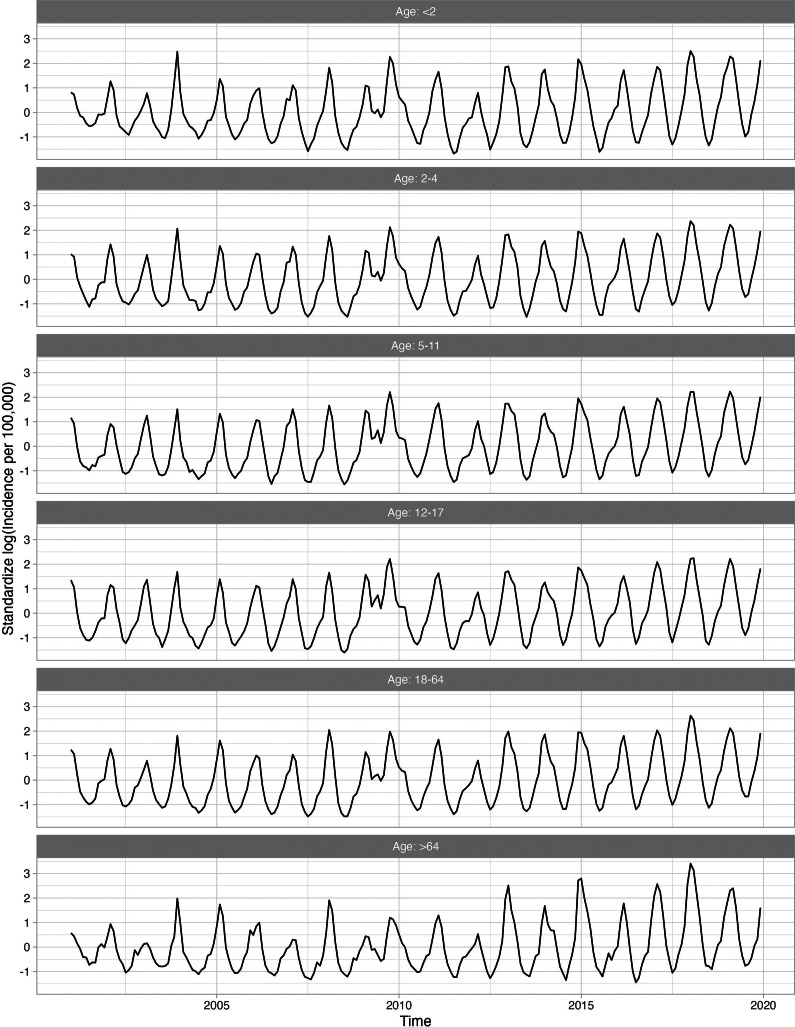
Standardized log-transformed influenza series for the age groups throughout the study period (2001–2019). A logarithmic transformation is applied to the incidence rate to produce an approximately stationary time series.

There were 208908470 distinct enrolees included from the Merative MarketScan Research Databases between 2001 and 2019. Of these enrolees, 7856928 had at least one influenza diagnosis during the study period, with a total of 9005956 distinct monthly influenza events that were used to construct six age-stratified analytical cohorts that spanned 19 influenza seasons. Table 1 provides baseline characteristics for this study population. Figure 1 plots the age-stratified incidence of identified influenza events each month.

**Table 1.** Baseline characteristics of the overall study population

	Entire study population
Start date	1 January 2001
End date	31 December 2019
Total population	208908470
Patients with ≥ 1 influenza event	7856928
Distinct monthly influenza events	9005956
Age at first enrolment (years)	
<2	9184263 (4.4%)
2–4	7944120 (3.8%)
5–11	18855294 (9.0%)
12–17	16969491 (8.1%)
18–64	144757645 (69.3%)
≥65	11197657 (5.4%)
Number of influenza events observed in each age group	
<2	225944
2–4	693342
5–11	1783527
12–17	1149456
18–64	4808733
≥65	335827
Female sex	107084675 (51.3%)

### Identification of influenza

**Figure 2.**
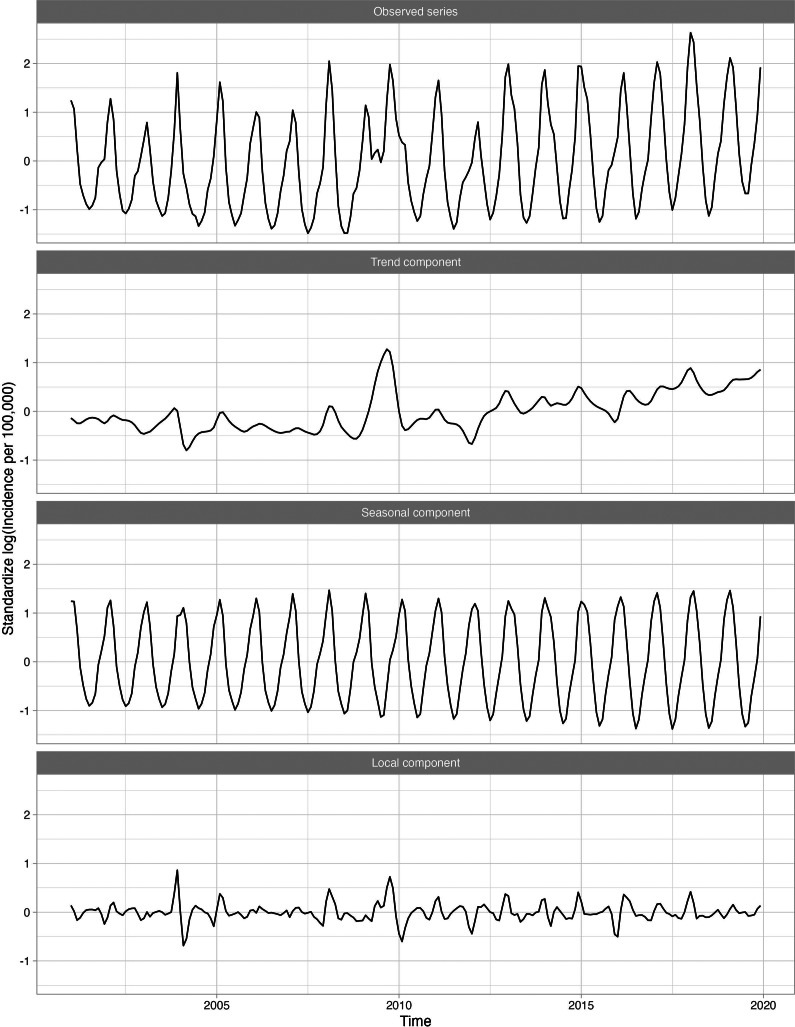
Standardized incidence per 100000 cases of influenza for 18–64 age group decomposed into the global trend, seasonal component, and anomaly and error component. A logarithmic transformation is applied to the incidence rate to produce an approximately stationary time series.

Figure 2 shows four standardized, log-transformed series corresponding to the influenza series for 18–64-year-olds, who were the largest age group evaluated. The trend component, representing the long-term trend in the influenza series, was relatively flat, indicating that influenza incidence did not increase over time. The seasonal component, representing the recurring seasonal pattern in the data, peaked every year around February. The local component identified the severe seasons which occurred in late 2003 and late 2009.

### Conditions positively associated with influenza

**Figure 3.**
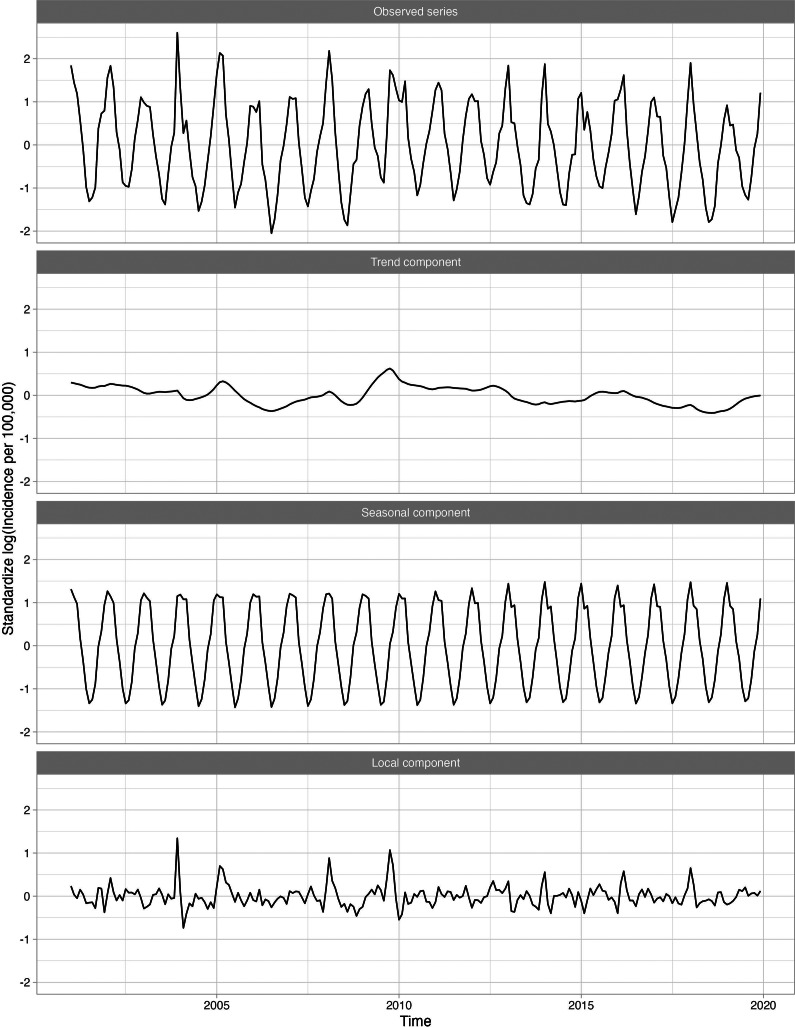
Standardized incidence per 100000 cases of pneumonia for 18–64 age group decomposed into the global trend, seasonal component, and anomaly and error component. A logarithmic transformation is applied to the incidence rate to produce an approximately stationary time series.

**Figure 4.**
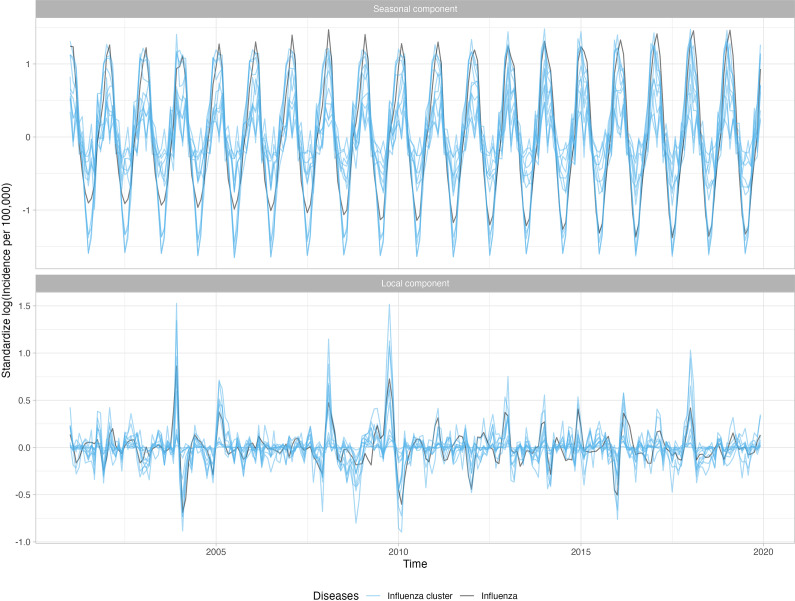
Standardized incidence per 100000 cases of influenza and the identified diseases associated with influenza for 18–64 age group decomposed into the seasonal component and anomaly and error component. A logarithmic transformation is applied to the incidence rate to produce an approximately stationary time series.

Overall, 23 of 282 (8.2%) diagnosis categories were highly associated with influenza activity for at least one age group. Supplementary Appendix Table 1 presents the disease categories that were positively related to influenza activity, along with their global and local correlations. The disease categories identified included both infectious (e.g., pneumonia, acute bronchitis, otitis media) and non-infectious diseases (e.g., myocardial infarctions, asthma). For example, the long-term trend for pneumonia (known to occur secondary to influenza) was relatively flat, while the seasonal component peaked in the winter with transitory peaks in late 2003 and late 2009, following the influenza series (Figure 3). Other series, including respiratory infections such as acute bronchitis, otitis media, and shock, were also seasonal and peaked in winter months, similarly to influenza. Figure 4 shows the 11 diseases for individuals aged 18–64, in which the seasonal and local time-series components for these diseases overlap with the influenza-derived components. More specifically, the identified influenza-cluster diseases not only have seasonal peaks that align with influenza but also have transitory peaks in late 2003 and late 2009, along with other spurious peaks, that track the incidence pattern of influenza.

Fever of unknown origin was the only CCS disease category that was globally and locally correlated with influenza across all age groups. Children 5–11 years of age had the highest number of disease conditions associated with influenza compared to the other age groups. Secondary diseases like viral infection, pneumonia, acute bronchitis, other upper respiratory infections, other lower respiratory diseases, and fever of unknown origin were all locally correlated with influenza for all groups ≥5 years of age. Acute myocardial infarction and cardiac arrest/ventricular fibrillation were only locally correlated with influenza in the ≥65 year age group, while COPD and bronchiectasis was locally correlated with influenza for all groups with people who were 5–64 years of age. Fever of unknown origin and malaise and fatigue were the only diseases locally correlated for children <2 years of age.

### Diseases negatively associated with influenza

**Figure 5.**
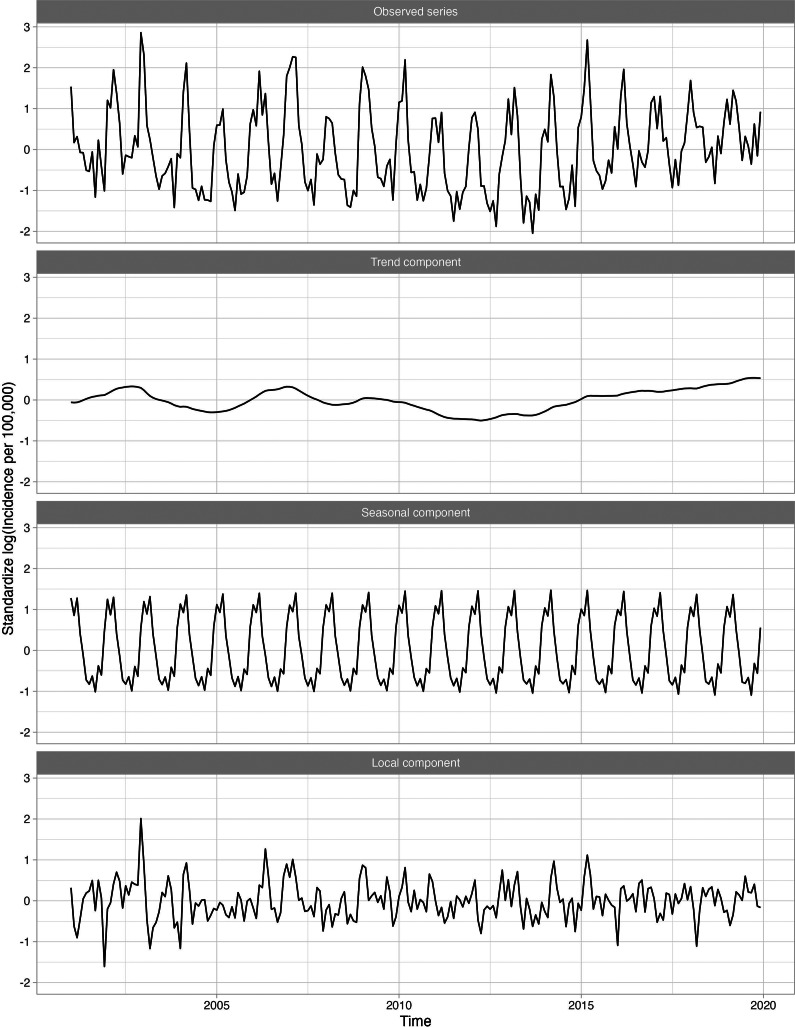
Standardized incidence per 100000 cases of non-infectious gastroenteritis for the 18–64 group decomposed into the global trend, seasonal component, and anomaly and error component. A logarithmic transformation is applied to the incidence rate to produce an approximately stationary time series. This secondary disease is an example of a disease that is globally correlated with influenza; however, is not locally correlated with influenza.

Some series are positively seasonally correlated with influenza but not locally. The series for non-infectious gastroenteritis, for example, was globally and seasonally, but not locally, correlated with influenza (Figure 5). For the 18–64-year age group, the global correlation was 0.895 while the local correlation was near zero (−0.063). This disease exhibited a local peak in late 2002 but no other distinct peaks are evident, and none align with influenza. Thus, non-infectious gastroenteritis was unlikely to be a secondary infection after influenza. Furthermore, some series, such as HIV infection and hypertension were not seasonal. Other series, such as skin and subcutaneous tissue infections, fractures, and burns were negatively seasonally correlated with influenza and peaked in the summer months. A list of all series that are negatively seasonally correlated with influenza is given in Supplementary Appendix Table 2.

### Reduction of secondary disease burden associated with reduced influenza burden

Table 2 reports the percentage decrease in the number of cases of each disease, per 100000 people, if peak influenza rates declined by 20% or 60% [19]. For example, a 60% decrease in the number of influenza cases resulted in a 24–30% decrease in the number of acute bronchitis cases across age groups. Those ≥65 years of age had the largest reduction: pneumonia cases decreased an average of 16%, and upper respiratory infection cases decreased more than 17%. However, even a 20% decrease in the number of influenza cases has significant effects on secondary infections. We estimate that, on average, cases of acute bronchitis would decrease by 7%, pneumonia by 4%, and other upper respiratory infections by a minimum of 4% during the peak month of influenza activity.

**Table 2.** Percent reduction in incidence rate for diseases that were globally and locally correlated with influenza assuming 20% and 60% reductions in influenza incidence

		Attributable reduction in disease incidence during the peak influenza month, associated with a 20%, 60% reduction in influenza incidence					
		Age group					
CCS	Disease	<2	2–4	5–11	12–17	18–64	>64
**2**	Septicaemia (except in labour)		2.240%, 8.882%	1.648%, 6.595%			
**3**	Bacterial infection; unspecified site			3.881%, 15.003%	1.346%, 5.413%		
**7**	Viral infection		1.911%, 7.616%	2.148%, 8.532%	1.590%, 6.370%	1.006%, 4.065%	
**55**	Fluid and electrolyte disorders			2.691%, 10.598%			
**63**	Diseases of white blood cells			1.230%, 4.954%			
**84**	Headache; including migraine			0.936%, 3.789%			
**92**	Otitis media and related conditions		5.552%, 20.907%	4.117%, 15.854%	2.310%, 9.150%	2.056%, 8.178%	
**100**	Acute myocardial infarction						0.680%, 2.764%
**107**	Cardiac arrest and ventricular fibrillation						0.985%, 3.984%
**122**	Pneumonia (except that caused by tuberculosis or sexually transmitted disease)			5.290%, 20.001%	4.638%, 17.716%	3.693%, 14.316%	3.283%, 12.808%
**124**	Acute and chronic tonsillitis			1.748%, 6.985%	1.633%, 6.537%		
**125**	Acute bronchitis			6.506%, 24.137%	6.849%, 25.273%	6.579%, 24.380%	8.297%, 29.930%
**126**	Other upper respiratory infections		4.386%, 16.821%	5.154%, 19.529%	5.642%, 21.216%	5.093%, 19.316%	6.979%, 25.701%
**127**	Chronic obstructive pulmonary disease and bronchiectasis			5.848%, 21.919%	6.031%, 22.543%	2.599%, 10.248%	
**128**	Asthma					0.992%, 4.013%	
**129**	Aspiration pneumonitis; food/vomitus						1.119%, 4.516%
**130**	Pleurisy; pneumothorax; pulmonary collapse			1.835%, 7.324%		0.516%, 2.104%	0.730%, 2.964%
**131**	Respiratory failure; insufficiency; arrest (adult)			1.141%, 4.605%		0.531%, 2.164%	1.320%, 5.309%
**133**	Other lower respiratory disease		5.308%, 20.064%	4.630%, 17.690%	4.010%, 15.468%	2.192%, 8.699%	1.244%, 5.010%
**246**	Fever of unknown origin	1.824%, 7.280%	3.894%, 15.050%	5.392%, 20.358%	6.082%, 22.715%	3.805%, 14.727%	1.953%, 7.781%
**247**	Lymphadenitis				1.029%, 4.158%		
**250**	Nausea and vomiting			3.372%, 13.137%	2.608%, 10.282%		
**252**	Malaise and fatigue	0.889%, 3.602%	1.397%, 5.615%	2.161%, 8.580%	1.795%, 7.167%		

Table 3 provides a breakdown of these cost estimates for each age group, extrapolated to the entire population. Supplementary Appendix Tables 3 and 4 provide additional cost breakdowns from which these estimates were obtained. Overall, we estimated that a 20% reduction in peak influenza incidence would be associated with a decrease in total direct medical costs of $1.8 billion (95% CI: 1.6–2.1), of which $1.4 billion (95% CI: 1.2–1.6) was due to secondary conditions associated with influenza. Similarly, a 60% reduction would be associated with a total medical cost decrease of $6.4 billion (95% CI: 6.0–6.8), of which $5.3 billion (95% CI: 4.9–5.7) was attributable to secondary conditions.

**Table 3.** Total cost reductions associated with visits for both influenza and secondary conditions are summarized in the left column, and cost reductions associated only with visits for secondary conditions are summarized in the right column

Age group	Total cost reduction (Influenza + Secondary conditions)		Cost reduction for secondary conditions alone	
	Per enrolee	Population total	Per enrolee	Population total
**20% reduction in peak influenza incidence**				
<2	3.05 (2.12–4.36)	21399806 (14866806–30593791)	0.68 (0.27–1.54)	4769249 (1920191–10773987)
2–4	8.35 (7.22–9.6)	95118089 (82273188–109299600)	6.15 (5.2–7.42)	70029905 (59242543–84511029)
5–11	5.33 (4.74–6)	152738938 (135901419–171896794)	3.58 (3.09–4.19)	102646509 (88442134–120108862)
12–17	3.12 (2.7–3.58)	81214290 (70305189–93219073)	2.05 (1.69–2.52)	53468418 (44047797–65765152)
18–64	3.24 (3.01–3.46)	655805473 (610524224–699846881)	2.49 (2.31–2.7)	505064760 (466997878–546219636)
>64	14.6 (12.22–17.07)	814781042 (681532533–952307959)	11.79 (9.39–14.31)	658034320 (523807811–798178690)
**Total**		**1821057638** **(1595403359–2057164098)**		**1394013161** **(1184458354–1625557356)**
**60% reduction in peak influenza incidence**				
<2	9.58 (7.98–11.25)	67161434 (55979286–78874858)	2.65 (1.74–4)	18557947 (12208227–28021271)
2–4	28.51 (26.64–30.49)	324685733 (303324594–347176011)	22.55 (20.77–24.48)	256777585 (236499402–278752912)
5–11	17.44 (16.59–18.32)	499743502 (475241473–524743460)	12.9 (12.04–13.82)	369497018 (344999868–395988030)
12–17	10.44 (9.74–11.14)	271900215 (253892077–290183070)	7.55 (6.83–8.27)	196589391 (177992581–215599723)
18–64	11.49 (11.11–11.87)	2327056329 (2249357271–2403462008)	9.47 (9.1–9.86)	1918569454 (1842467691–1998123563)
>64	52.55 (48.23–57.18)	2931742126 (2690760235–3190456755)	44.93 (40.63–49.57)	2506690470 (2267036152–2765507906)
**Total**		**6422289339** **(6028554936–6834896162)**		**5266681865** **(4881203921–5681993405)**

## Discussion

In this time-series analysis, we identified multiple disease categories both globally and locally correlated with influenza activity. The disease categories included both infectious (e.g., pneumonia, acute bronchitis, and otitis media) and non-infectious diseases (e.g., myocardial infarctions, asthma, and COPD), and the nature of the relationship varied by age. Identifying the specific disease categories that were strongly associated with influenza allowed us to explore how decreases in influenza activity could potentially decrease the burden and healthcare costs attributable to secondary diseases. Collectively, our results demonstrate how ignoring secondary diseases associated with influenza may lead to substantial underestimates of the overall burden of influenza. This finding provides an additional rationale to increase influenza vaccination rates and non-pharmacological interventions to reduce the risk of primary influenza infections.

Time-series approaches have been used in a wide variety of applications [20, 21] to demonstrate a temporal association between two series that are hypothesized to be causally related. While time-series approaches cannot directly establish causality, the presence of both a global and a local temporal relationship provides evidence in support of potential causation. While time-series approaches have been used to study the association of influenza with other diseases [12–14], unlike in prior work, we did not prespecify a set of diseases that could be associated with influenza. In contrast, we considered the entire set of CCS codes rather than focusing on a single disease.

The plausibility of our time-series results is supported by prior literature for many of the disease conditions we identified. For example, the association between influenza and bacterial pneumonia has been described in clinical studies [22] and animal models [23]. Multiple mechanisms may play a role in increasing the risk for bacterial pneumonia after influenza, including changes in mucociliary clearance and cellular immunity [23, 24]. Evidence for the association between influenza and bacterial otitis media also exists from animal models [8, 9], human influenza challenge studies [25], and studies of influenza vaccination [26]. In addition, clinical reports have associated influenza with bronchitis and other respiratory infections, including tonsilitis [27]. In the case of asthma, exacerbations are thought to be attributable to influenza infections causing airway hyperresponsiveness [14]. For COPD, influenza-associated exacerbations may be caused by airway hyperresponsiveness or secondary bacterial infections [13].

Most of the prior cost-related-influenza literature focuses on either estimating the healthcare costs associated with cases of influenza alone or combined with pneumonia [28]. In general, cost estimates vary widely because measurement approaches have differed substantially across studies [29]. Some studies include only healthcare-associated expenses [28], some include lost productivity in the workforce, while others consider losses associated with mortality [7, 30]. However, to date, most efforts do not include a broad range of secondary diseases beyond pneumonia. Accordingly, our cost estimates are not directly comparable to other studies, but they do highlight how prior studies likely underestimate the full economic burden of influenza. For example, we estimated cost savings of $1.6 billion from a 20% influenza reduction, 76% of which was due to secondary healthcare, and $6.4 billion from a 60% influenza reduction, 82% of which was due to secondary healthcare.

Some of the 23 conditions we identified (e.g., viral infections, other respiratory infections) may also capture undiagnosed cases of influenza rather than secondary respiratory infections, because cases of influenza are commonly underdiagnosed [31]. In addition, a few of the different respiratory conditions identified by our analysis (e.g., other lower respiratory infections, respiratory failure) could either be attributable to undiagnosed cases of influenza [32] or secondary bacterial infections. Nevertheless, even if some of the potential secondary conditions that we identified represent cases of influenza, our estimates still help address the underestimates of disease burden and cost associated with prior studies.

The CCS code ‘fever of unknown origin’ was the only disease condition highly associated with influenza across all age groups. Influenza is not typically a cause of fever of unknown origin. Fevers of unknown origin refers to prolonged fevers without a clear aetiology despite comprehensive evaluation [33]. However, the CCS code for ‘fever of unknown origin’ includes the diagnostic code for ‘fever unspecified’, which does not require a specific duration or level of diagnostic investigation. Thus, it is possible that the fever of unknown origin finding was driven by the fact that fever is a common symptom. Also, biphasic fevers [34], two distinct periods of fever – one from the initial influenza infection and one from a secondary bacterial infection – may increase the probability of an ‘unspecified fever’ diagnosis.

Other CCS categories varied across age groups. Some of the variation is likely based on the epidemiology of different disease categories. For example, it was not surprising that acute myocardial infarctions were only identified in people over 65 years of age. In contrast, we found the CCS code for COPD and bronchiectasis was not identified in people over 65 years old. This was unexpected. It is unlikely that older people with COPD and bronchiectasis are less vulnerable to the effects of influenza, instead they may have been diagnosed with a pulmonary disease or respiratory infection not included in the COPD and bronchiectasis CCS code set.

Several disease categories were only identified in paediatric populations. For example, ‘malaise and fatigue’ was highly correlated with influenza only in those <18 years. Furthermore, ‘fluid and electrolyte disorders’, ‘diseases of white blood cell’ and ‘headache’ were only identified in those 5–11 years of age. In contrast, several conditions were identified only in adults: ‘pleurisy, pneumothorax, pulmonary collapse’ and ‘respiratory failure; insufficiency; arrest’. Some age-related differences may represent variations in immune responses that occur across the lifespan [35]. However, some differences are likely reflective of diagnostic and coding practices or the aggregation of multiple different codes into a single CCS code.

A major limitation associated with this work is our use of CCS codes, which are commonly used to transform a large number of diagnostic codes into a smaller set of categories [36]. This approach allowed us to explore a comprehensive list of disease categories. However, with CCS codes we lost the ability to explore some conditions that lack a specific CCS category but have been previously linked to influenza. Examples of such diseases potentially attributable to influenza include *Clostridioides difficile* infection [37], myocarditis, encephalitis, Guillian-Barre syndrome, and Reye’s syndrome [38].

In addition to the use of CCS codes, our work has several additional limitations. First, we exclusively used administrative data and do not have test results or clinical notes to validate the diagnoses considered. Second, only patients with private insurance or supplemental Medicare coverage are included. People without private insurance may have different health-seeking behaviours. Third, our analysis is ecological. We studied the ‘correlation’ with influenza activity to investigate influenza’s association with each CCS code. Thus, we are not able to determine if individuals with these CCS codes suffered from influenza during the exposure period. However, this is also an advantage to our approach, as influenza is often undiagnosed, and many people who may not seek care for influenza may seek care for the CCS codes considered. Fourth, it is possible that other viral respiratory pathogens that circulate around the same time as influenza (e.g., respiratory syncytial virus, parainfluenza virus, or human metapneumovirus) could contribute to some of our overall disease-burden findings. Finally, we did not consider the effects of comorbidities or influenza-vaccination status. Accordingly, our estimates should be viewed as rough approximations, and future work will be needed to provide more precise estimates.

In conclusion, we developed a data-driven approach to identify secondary diseases attributable to influenza. Our approach did not require prespecifying specific diseases, allowing us to estimate more broadly how much secondary diseases could be reduced if influenza activity could be reduced. Our findings suggest that the disease burden and costs attributable to influenza are substantially underestimated if secondary diseases are not considered. Future work is needed to extend our approach using more granular measures of secondary diseases and conditions associated with influenza.

## Supporting information

10.1017/S0950268826101320.sm001 Miller et al. supplementary material Miller et al. supplementary material

## Data Availability

The data that support the findings of this study are available from Merative Marketscan. Restrictions apply to the availability of these data, which were used under license for this study.

## Supplementary material

The supplementary material for this article can be found at http://doi.org/10.1017/S0950268826101320.
