# Supplementary material for: Disease burden associated with influenza activity at the population level
Source: Epidemiol Infect. 2026 Apr 6;154:e53. doi: 10.1017/S0950268826101320 (PMC13161811; doi:10.1017/S0950268826101320)
Supplement: Miller et al. supplementary material [file S0950268826101320sup001.docx]

**APPENDIX**

***Appendix Methods 1*** – Modeling disease-incidence series, identifying secondary diseases related to influenza, and calculating reduction incidence attributable to a target reduction in influenza.

*Constructing state-space time-series models for disease-incidence series and identifying casually related secondary diseases*

For each age-stratified incidence series, we used a structural (state-space) time-series model[1, 2] that allowed us to decompose each series into three meaningful additive components: a long-term trend component, a recurring seasonal component, and a component that represents residual white noise, in addition to an anomaly process that characterizes local temporal correlation based on transitory peaks and troughs. For brevity, we refer to the additive combination of the anomaly and noise processes as the local component.

We note that three components are needed for the identification of secondary diseases associated with influenza, because two series may be spuriously correlated if they simply exhibit similar trends or seasonal patterns. The local component allows us to capture deviations from the baseline incidence level, which is established by the combination of the trend and the seasonal pattern that suggests atypically high or low incidence. Series with correlated local components are more likely to be causally related.

Before fitting the model, the claims series were log-transformed and standardized. The log transformation was needed to attenuate heteroscedasticity and make the assumption of stationarity more defensible for the detrended series. The standardization was performed so we could compare different series that have various levels of incidence. Our modeling and clustering approach is similar to the algorithm developed in Bengtsson and Cavanaugh (2008).[3]

The general specification for the proposed structural model is as follows:

$$Y_{t}=X_{t,1}+X_{t,2}+X_{t,3}+v_{t}; X_{t,1}=X_{t-1,1}+w_{1t}; X_{t,2}=-\sum_{j=1}^{12-1} X_{t-j,2}+w_{2t},$$

where the outcome variable $Y_{t}$ is the log-transformed, standardized monthly incidence of diagnosis claims. The three latent structures are represented by $X_{t,1}$, $X_{t,2}$, and $X_{t,3}$, respectively. We employ a random-walk model to characterize the long-term trend $X_{t,1}$, since such a model will accommodate linear as well as nonlinear changes over time. The seasonal model for $X_{t,2}$ has observations summed to a zero-mean disturbance term over the course of the year, which would be reflected in sinusoidal patterns. The local anomaly component $X_{t,3}+ v_{t}= Y_{t}-X_{t,1}-X_{t,2}$ is obtained from the observed incidence series once the long-term trend and seasonal pattern are extracted. Although in our analytic framework, the anomaly component $X_{t,3}$ is not explicitly modeled, this process could be conceptualized as representing transitory temporal associations that persist for recent months after removal of the baseline trend and seasonality. The white noise term $v_{t}$ represents the variability in the observed incidence series that is unexplained by the two structural components. The terms $w_{1t}$ and $w_{2t}$ represent the white noise terms that are used in defining the corresponding latent processes of the model. Each white noise series is comprised of independent, identically distributed variates that follow a zero-mean normal distribution with a constant variance.

Based on the corresponding seasonal and local components extracted from the age-stratified disease series, we applied a two-step, model-based screening method to find diagnosis series that are both globally and locally correlated with influenza given age. First, we identified those diagnosis series that are seasonally correlated with influenza by comparing the extracted seasonal component for each age-specific diagnosis series to that of the influenza series. The diagnosis series was deemed as globally clustered with the influenza series if the correlation between the seasonal components was 0.6 or higher. Second, within the global influenza cluster, we identified the diagnosis series that are locally correlated with influenza by comparing the local (anomaly plus noise) components. Specifically, diagnosis series were deemed as locally clustered with the influenza series if the correlation between the local components were greater than or equal to 0.2.

*Translating different levels of influenza activity with healthcare utilization and costs of the burden of post-acute sequalae.*

In order to determine the real-world effects of influenza for each age group, we used those series that were found to be both globally and locally associated with influenza to build time-series-forecasting models. We then used these models to predict the reduction in incidence for each diagnostic class associated with different levels of influenza attenuation. Each forecasting model is formulated as a time series regression of the following form:

$$y_{t}=\beta_{0}+\beta_{1}x_{t}+e_{t}$$

where $y_{t}$ is the detrended outcome series (seasonal plus local secondary disease components), $x_{t}$ is the detrended monthly influenza series, and $e_{t}$ is a white-noise series representing the unexplained variation in the diagnosis series${, y}_{t}$,not explained by the influenza series. Using this time-series model, we then evaluated the real-world effects of changing levels of influenza on each secondary outcome, including healthcare utilization and costs.

To estimate the real-world effects of changing levels of influenza on each age-stratified secondary disease outcome, the maximum mean monthly incidence (i.e., peak month) for influenza was reduced by 20% and 60% (i.e., target reduction rates), which demonstrates the possible effectiveness of vaccines, increased uptake of influenza vaccines and treatments, or non-pharmaceutical interventions.[4]. With the peak-month incidence and target-reduction incidence for influenza, the reduction percentage from the peak-secondary-disease outcome was predicted. Thus, the model provides an estimate of attributable reduction in the secondary-disease incidence due to a decrease in influenza incidence.

***Appendix Methods 2*** – Estimating healthcare visit costs attributable to a targeted reduction in influenza incidence.

We developed a cost simulation model to estimate the reduction in healthcare-visit costs attributable to either a 20% or 60% reduction in influenza incidence. Similar to the analysis of the attributable secondary-disease burden, we focus on the peak month of influenza. Specifically, we use data for the month of February 2019, which represented the peak month of influenza activity during 2019. We also focus on costs associated with healthcare visits and do not account for medication costs that may also occur along with these visits. *Thus, our cost analysis represents a lower bound on the overall healthcare burden that may be preventable with a reduction in influenza incidence.*

Two considerations must be addressed when computing healthcare costs associated with the various secondary conditions we identified. First, the secondary-disease burden reflected by the 23 conditions outlined in Table 5 are highly correlated and multiple conditions may occur within the same healthcare encounter. For example, a single healthcare encounter might involve a diagnosis of bacterial infection (CCS 3), pneumonia (CCS 122), headache (CCS 84) and malaise and fatigue (CCS 252). Thus, one cannot simply sum the costs associated with the healthcare visits for each of these conditions, insofar as this will result in double counting visits representing concomitant conditions. Second, healthcare received for a given condition, such as pneumonia, may involve care received during multiple encounters. Thus, one must include all healthcare encounters during an episode of care for a given condition.

To address these two considerations, we developed a cost-simulation model to bootstrap the secondary-disease burden. The following is a detailed summary of this algorithm:

1. Draw a bootstrapped sample of patients for each age group and CCS code based on the corresponding attributable rates outlined in Table 5. For example, to compute the cost reduction attributable to a 20% reduction in influenza for age group <2, we would randomly draw 20% of these patients who had an influenza encounter, 1.824% who had a fever encounter (CCS 246) and 0.889% who had a visit for malaise and fatigue (CCS 252). For each of these patient-disease pairings selected, we then collect all visits during the peak month for the given condition.
2. Reduce the set of patient visits selected to only the distinct set of patient visits (i.e., remove repeated visits that contain multiple identified CCS codes).
3. Sum the costs of the visits that were retained in step (2) for each patient age group.
4. Repeat steps (1)-(3) 1000 times
5. Generate median point estimates and percentile-based CIs across the set of cost estimates generated from step (4)
6. Scale to the population using census weights and enrollment within each age group. First, we compute the number of enrollees in each age group that are represented during the peak-influenza month. We then divide the cost estimates by the number of enrollees to derive an estimate of cost per enrollee. Finally, we multiple the per-enrollee costs by the overall US population in each specific age bin.

This procedure was repeated for both a 20% and a 60% decline in peak influenza incidence. Appendix Tables 1 and 2 provide the specific cost estimates and census- population values that were used to derive these estimates.

***Appendix Table 1****--* Global and local correlation values for conditions that met correlation cut-points by age group. Diseases with missing correlation values are an indication that for the age group of interest the disease was not globally and locally correlated with influenza

|  | | **Age group** | | | | | |
| --- | --- | --- | --- | --- | --- | --- | --- |
| **CCS** | **Disease** | **<2** | **2-4** | **5-11** | **12-17** | **18-64** | **>64** |
|  |  | *Global, local correction value* | | | | | |
| **2** | Septicemia (except in labor) |  | 0.746, 0.206 | 0.864, 0.282 |  |  |  |
| **3** | Bacterial infection; unspecified site |  |  | 0.899, 0.241 | 0.786, 0.376 |  |  |
| **7** | Viral infection |  | 0.752, 0.709 | 0.929, 0.775 | 0.825, 0.771 | 0.768, 0.766 |  |
| **55** | Fluid and electrolyte disorders |  |  | 0.861, 0.450 |  |  |  |
| **63** | Diseases of white blood cells |  |  | 0.751, 0.287 |  |  |  |
| **84** | Headache; including migraine |  |  | 0.652, 0.370 |  |  |  |
| **92** | Otitis media and related conditions |  | 0.937, 0.244 | 0.921, 0.392 | 0.838, 0.459 | 0.916, 0.334 |  |
| **100** | Acute myocardial infarction |  |  |  |  |  | 0.603, 0.377 |
| **107** | Cardiac arrest and ventricular fibrillation |  |  |  |  |  | 0.656, 0.303 |
| **122** | Pneumonia (except that caused by tuberculosis or sexually transmitted disease) |  |  | 0.912, 0.303 | 0.925, 0.590 | 0.975, 0.696 | 0.913, 0.638 |
| **124** | Acute and chronic tonsillitis |  |  | 0.718, 0.417 | 0.923, 0.427 |  |  |
| **125** | Acute bronchitis |  |  | 0.911, 0.549 | 0.915, 0.706 | 0.948, 0.708 | 0.941, 0.624 |
| **126** | Other upper respiratory infections |  | 0.939, 0.370 | 0.937, 0.643 | 0.915, 0.671 | 0.961, 0.549 | 0.944, 0.487 |
| **127** | Chronic obstructive pulmonary disease and bronchiectasis |  |  | 0.906, 0.559 | 0.917, 0.702 | 0.935, 0.669 |  |
| **128** | Asthma |  |  |  |  | 0.743, 0.364 |  |
| **129** | Aspiration pneumonitis; food/vomitus |  |  |  |  |  | 0.733, 0.341 |
| **130** | Pleurisy; pneumothorax; pulmonary collapse |  |  | 0.849, 0.339 |  | 0.708, 0.286 | 0.623, 0.315 |
| **131** | Respiratory failure; insufficiency; arrest (adult) |  |  | 0.708, 0.272 |  | 0.744, 0.409 | 0.810, 0.471 |
| **133** | Other lower respiratory disease |  | 0.946, 0.322 | 0.934, 0.640 | 0.937, 0.746 | 0.955, 0.671 | 0.769, 0.529 |
| **246** | Fever of unknown origin | 0.786, 0.787 | 0.873, 0.818 | 0.972, 0.875 | 0.981, 0.900 | 0.928, 0.902 | 0.788, 0.832 |
| **247** | Lymphadenitis |  |  |  | 0.826, 0.201 |  |  |
| **250** | Nausea and vomiting |  |  | 0.962, 0.378 | 0.877, 0.377 |  |  |
| **252** | Malaise and fatigue | 0.627, 0.274 | 0.875, 0.572 | 0.932, 0.585 | 0.740, 0.222 |  |  |

**Appendix Table 2** -- Selected global correlation values for diseases classified as peaking in the summer. A disease is classified as a summer disease by having a global correlation of -0.6 or lower (i.e., -1 times the global correlation threshold) with the seasonal component of influenza based on the age group. The diseases presented here have a correlation value of -0.9 or lower. A correlation value is missing if for the age group of interest, the correlation value was not less than -0.9

|  | | **Age group** | | | | | |
| --- | --- | --- | --- | --- | --- | --- | --- |
| **CCS** | **Disease** | **<2** | **2-4** | **5-11** | **12-17** | **18-64** | **>64** |
| **8** | Other infections; including parasitic |  | -0.927 |  |  |  |  |
| **121** | Other diseases of veins and lymphatics |  |  | -0.909 |  |  |  |
| **197** | Skin and subcutaneous tissue infections |  | -0.910 | -0.930 |  | -0.938 |  |
| **229** | Fracture of upper limb |  | -0.927 |  |  |  |  |
| **230** | Fracture of lower limb |  |  |  |  | -0.919 |  |
| **234** | Crushing injury or internal injury |  |  |  |  | -0.920 |  |
| **235** | Open wounds of head; neck; and trunk |  |  |  |  | -0.909 |  |
| **236** | Open wounds of extremities |  | -0.911 | -0.923 | -0.931 | -0.961 | -0.931 |
| **239** | Superficial injury; contusion | -0.950 | -0.922 |  |  | -0.938 | -0.924 |
| **240** | Burns |  |  |  | -0.94 | -0.930 |  |
| **243** | Poisoning by nonmedicinal substances | -0.911 | -0.929 | -0.957 | -0.932 | -0.938 |  |
| **2601** | E Codes: Cut/pierced |  |  |  |  | -0.910 |  |
| **2608** | E Codes: Pedal cyclist; not MVT |  |  |  |  | -0.925 |  |
| **2611** | E Codes: Natural/environment | -0.937 | -0.945 |  |  | -0.927 |  |

***Appendix Table 3*** – Results of simulation cost analysis for a 20% reduction in peak influenza incidence.

| **Age Group** | **Total Enrollee Months** | **Simulation cost estimate across all enrollees in age group** | **Per Enrollee Cost** | **2020 US Census Population** | **Total Population Cost** |
| --- | --- | --- | --- | --- | --- |
| <2 | 234,491 | 715,575  (497,122-1,023,007) | 3.05  (2.12-4.36) | 7,012,629 | 21,399,806  (14,866,806-30,593,791) |
| 2-4 | 689,607.5 | 5,760,135  (4,982,277-6,618,935) | 8.35  (7.22-9.6) | 11,387,606 | 95,118,089  (82,273,188-109,299,600) |
| 5-11 | 1,839,924.82 | 9,808,899  (8,727,593-11,039,217) | 5.33  (4.74-6) | 28,650,327 | 152,738,938  (135,901,419-171,896,794) |
| 12-17 | 1,853,351.07 | 5,776,859  (5,000,883-6,630,772) | 3.12  (2.7-3.58) | 26,055,438 | 81,214,290  (70,305,189-93,219,073) |
| 18-64 | 16,370,339.6 | 53,002,799  (49,343,127-56,562,266) | 3.24  (3.01-3.46) | 202,550,780 | 655,805,473  (610,524,224-699,846,881) |
| >64 | 901,266.86 | 13,161,897  (11,009,413-15,383,494) | 14.6  (12.22-17.07) | 55,792,501 | 814,781,042  (681,532,533-952,3079,59) |

***Appendix Table 4*** – Results of simulation cost analysis for a 60% reduction in peak influenza incidence.

| **Age Group** | **Total Enrollee Months** | **Simulation cost estimate across all enrollees in age group** | **Per Enrollee Cost** | **2020 US Census Population** | **Total Population Cost** |
| --- | --- | --- | --- | --- | --- |
| <2 | 234,491 | 2,245,770  (1,871,857-2,637,448) | 9.58  (7.98-11.25) | 7,012,629 | 67,161,434  (55,979,286-78,874,858) |
| 2-4 | 689,607.5 | 19,662,229  (18,368,647-21,024,189) | 28.51  (26.64-30.49) | 11,387,606 | 324,685,733  (303,324,594-347,176,011) |
| 5-11 | 1,839,924.82 | 32,093,542  (30,520,021-33,699,040) | 17.44  (16.59-18.32) | 28,650,327 | 499,743,502  (475,241,473-524,743,460) |
| 12-17 | 1,853,351.07 | 19,340,552  (18,059,614-20,641,031) | 10.44  (9.74-11.14) | 26,055,438 | 271,900,215  (253,892,077-290,183,070) |
| 18-34 | 16,370,339.6 | 188,074,824  (181,795,115-194,250,001) | 11.49  (11.11-11.87) | 202,550,780 | 2,327,056,329  (2,249,357,271-2,403,462,008) |
| >64 | 901,266.86 | 47,359,089  (43,466,290-51,538,341) | 52.55  (48.23-57.18) | 55,792,501 | 2,931,742,126  (2,690,760,235-3,190,456,755) |

(1) **Harvey AC.** Forecasting, structural time series models and the Kalman filter. 1990.

(2) **Shumway RH, Stoffer DS, Stoffer DS**. *Time series analysis and its applications*: Springer, 2000.

(3) **Bengtsson T, Cavanaugh JE.** State‐space discrimination and clustering of atmospheric time series data based on Kullback information measures. *Environmetrics: The official journal of the International Environmetrics Society* 2008; **19**(2): 103-121.

(4) Centers for Disease Control and Prevention. Vaccine Effectiveness: How Well Do Flu Vaccines Work? Questions and Answers. In, 2023.
